# Supplementary material for: Molecular docking and MD simulations predicted quercetin as a potent human interleukin-1 beta (hIL1β) inhibitor for improved endodontic disease management
Source: PLoS One. 2025 Dec 10;20(12):e0336841. doi: 10.1371/journal.pone.0336841 (PMC12694838; doi:10.1371/journal.pone.0336841)
Supplement: Attachment — Submitted filename: Reviwer report.docx [file pone.0336841.s001.docx]

I have carefully reviewed the manuscript titled "Quercetin as a Potent IL-1β Inhibitor: Molecular Docking and MD Simulation Insights for Improved Endodontic Disease Management". Below are my detailed comments and suggestions aimed at improving the scientific quality and clarity of the manuscript.

1- The title and scope of the manuscript suggest a novel investigation into the potential of quercetin as an IL-1β inhibitor; however, the authors appear to have overlooked a substantial body of existing literature that has already established this association. Quercetin has been widely reported as a potent IL-1β inhibitor in various models and disease contexts. For instance:

- *Quercetin Inhibits the Production of IL-1β-Induced Inflammatory Cytokines and Chemokines in ARPE-19 Cells via the MAPK and NF-κB Signaling Pathways.*
- *Regulation of IL-1-Induced Selective IL-6 Release from Human Mast Cells and Inhibition by Quercetin.*
- *Quercetin Inhibits IL-1β-Induced Proliferation and Production of MMPs, COX-2, and PGE2 by Rheumatoid Synovial Fibroblasts.*
- *Quercetin Reduces Inflammatory Pain: Inhibition of Oxidative Stress and Cytokine Production.*

Given this, the study lacks sufficient novelty, as it does not clearly differentiate itself from prior work or explain how it advances the current understanding of quercetin’s anti-inflammatory mechanisms. The authors are encouraged to justify the originality of their study and better position it within the existing literature.

2- The stated aim of the manuscript—"to identify effective treatments that can directly target the inflammatory processes central to PTED" by exploring the impact of flavonoids such as quercetin on IL-1β—is not sufficiently justified in terms of novelty. While the therapeutic targeting of IL-1β in inflammatory conditions is indeed important, quercetin's anti-inflammatory and IL-1β inhibitory activities are already well-documented across multiple studies and disease models. Therefore, presenting quercetin as a novel candidate in this context appears redundant.
3- To strengthen the manuscript, the authors should clearly delineate what is new in their study.
4- The authors report using SWISS-PDB Viewer for both protein and ligand preparation. However, this tool is primarily intended for visualizing and optimizing protein structures, particularly for tasks such as energy minimization of amino acid residues or modeling mutations. It is not suitable or widely accepted for ligand optimization or ligand geometry.
5- The methodology lacks clarity regarding the assignment of partial charges to the ligand molecules. It is unclear whether Gasteiger charges were correctly assigned using AutoDock Tools, as the text suggests that ligand preparation may have been conducted solely using SWISS-PDB Viewer, which is not suitable for this purpose. Accurate partial charge assignment is a critical step in molecular docking protocols, particularly when using AutoDock, which relies on Gasteiger charges for calculating interaction energies.
6- The selection of quercetin based on a slightly more negative docking score (–10.3 kcal/mol) compared to the co-crystallized ligand (–9.5 kcal/mol) is not sufficient, as the difference is within AutoDock Vina’s error range. Moreover, the manuscript lacks a clear filtering threshold; typically, a meaningful selection should involve compounds scoring at least –2 kcal/mol lower than the reference ligand to indicate significant improvement. The authors should justify the selection more rigorously and provide a comparative summary of the top-ranked compounds.
7- The discussion is shallow, and key tables (e.g., Table 2) lack clarity and need revision. The manuscript lacks novelty, presents redundant findings, and does not offer a logical or compelling scientific narrative. I recommend **rejection** in its current form and urge the authors to revise the work thoroughly with a clearer focus and stronger justification.
