# Supplementary material for: Molecular docking and MD simulations predicted quercetin as a potent human interleukin-1 beta (hIL1β) inhibitor for improved endodontic disease management
Source: PLoS One. 2025 Dec 10;20(12):e0336841. doi: 10.1371/journal.pone.0336841 (PMC12694838; doi:10.1371/journal.pone.0336841)
Supplement: Attachment — Submitted filename: Author_Response_to_Reviewer_Comments_auresp_2.docx [file pone.0336841.s004.docx]

**Author Response to Editor and Reviewer Comments**

Manuscript ID: PONE-D-25-25892

Molecular Docking and MD Simulations Predicted Quercetin as a Potent Human Interleukin-1 Beta (hIL1β) Inhibitor for Improved Endodontic Disease Management

**Editor Comments:**

**Editor Comment 1:** I suggest that authors maintain the 100ns md simulation time, but make the new modifications requested by the referee.

**Author response:** Dear Editor thank you very much for the valuable suggestion. In accordance with your recommendation, we have maintained the 100 ns molecular dynamics (MD) simulation time and incorporated the new modifications as requested. These adjustments have been carefully implemented, and the simulation results have been updated accordingly to reflect the improvements.

**Editor Comment 2:** Furthermore, all graphs in the manuscript should be in ns.

Author response: Dear Editor thank you very much for the valuable suggestion. We would like to bring into your kind information that the graphs presented in the manuscript have been incorporated directly as they are the standard output generated by the analysis package we used for data processing. These outputs reflect the original data representation without manual alteration, ensuring accuracy and reproducibility of the results.

**Reviewer comments:**

**Reviewer #3:**

**Reviewer Comment 1:** In the revised manuscript also, no novel concept is utilized while analyzing the results in support of IL-1β inhibition. Only the conventional techniques are used to explain the well-known phytochemical Quercetin and hIL-1β interactions without the sufficient statistical analyses. The manuscript still lacks the following things, most of which were already suggested for its improvement.

**Author response:** We thank the reviewer for the insightful and constructive comments. We would like to address the concerns raised as follows:

Regarding Novelty of Concept, our study focuses on a systematic in silico investigation of the interaction between the phytochemical Quercetin and human IL-1β, aiming to provide deeper mechanistic insights into their binding behavior and potential inhibitory effects. While the individual techniques used, such as molecular docking and molecular dynamics simulations, are well-established, the novelty of our work lies in the integrative approach combining multiple computational analyses under carefully defined conditions. We have revised the manuscript to highlight this integrated in silico strategy as a key contribution, emphasizing the specific binding patterns, interaction energy calculations, and dynamic stability analyses that strengthen the understanding of Quercetin’s potential inhibitory mechanism. The revised manuscript now more clearly presents how our in silico findings provide useful predictive data to guide further experimental validation of Quercetin as an IL-1β inhibitor. We appreciate the reviewer’s valuable suggestions, which have significantly improved the clarity, rigor, and impact of our manuscript.

**Reviewer Comment 2:** The data have been presented only from the short (100 ns) simulations though the limitations are written in the Discussion section. The ligand could escape away from the binding pocket in longer simulations. Therefore, it needs further analyses to evaluate the system stability. The protein-ligand relative stability tests (such as RDF plots, or distance plots of H-bonding atom pairs), intermediate protein-ligand snapshots during simulation, and active residue contributions to the MM/PBSA free energy should be described.

**Author response:** Respected reviewer, thank you for this observation. We acknowledge that the 100 ns simulation may be insufficient to capture full conformational stability. Due to computational constraints, we limited the simulation to 100 ns but have now addressed this limitation in the revised manuscript. We have also incorporated MM/PBSA free energy calculations based on the 100 ns trajectory in the revised manuscript to strengthen the analysis.

**Reviewer Comment 3:** The units are inconsistent, e.g. kj/mol should be kJ/mol in table 5. There should be the same unit for the same physical quantity throughout the manuscript: kJ/mol or kcal/mol? ns or ps? Make them uniform in figures, tables and main text.

**Author response:** We thank the reviewer for the valuable comment. We have carefully reviewed the entire manuscript, including all tables, figures, and the main text, to ensure consistency of the units used, wherever appropriate. All energy units have been standardized to **kJ/mol in the table 5.** Table 5 and other relevant sections have been corrected accordingly.

**Reviewer Comment 4:** The figures 1-4 are of poor quality. The table 2 text is not properly formatted. The y-axis title of figure 5 is cropped or unclear. The color representations are also unclear in figure 5. The legend texts in figure 1A-F are not readable.

**Author response:** We thank the reviewer for the careful examination of our figures and table, and for the valuable feedback. We have taken the following actions to address the concerns: The resolution and overall quality of Figures 1 through 4 have been improved to ensure clarity and readability in the revised manuscript. The formatting of Table 2 has been carefully corrected to ensure proper alignment, consistent font size, and appropriate spacing, improving its overall readability. The y-axis title in Figure 5 has been adjusted to avoid cropping and is now fully visible. Additionally, the color scheme has been defined to differentiate of data elements, improving visual interpretation. The text in the legends of Figure 1A-F has been enlarged and reformatted to enhance legibility.
