# Supplementary material for: Molecular docking and MD simulations predicted quercetin as a potent human interleukin-1 beta (hIL1β) inhibitor for improved endodontic disease management
Source: PLoS One. 2025 Dec 10;20(12):e0336841. doi: 10.1371/journal.pone.0336841 (PMC12694838; doi:10.1371/journal.pone.0336841)
Supplement: Attachment — Submitted filename: Author Response to Reviewer Comments-28-10-2025.docx [file pone.0336841.s005.docx]

**Author Response to Editor and Reviewer Comments**

Manuscript ID: PONE-D-25-25892R2

Molecular Docking and MD Simulations Predicted Quercetin as a Potent Human Interleukin-1 Beta (hIL1β) Inhibitor for Improved Endodontic Disease Management

**Editor Comments:**

**Editor Comment 1:** All graphics must be in ns.

**Author response:** Respected Editor, we have updated all graphics to use ns as the time unit to maintain consistency throughout the manuscript.
